# FUTURE PERSPECTIVES IN MELANOMA RESEARCH. Meeting report from the “Melanoma Research: a bridge from Naples to the World. Napoli, December 5^th^–6 ^th^2011”

**DOI:** 10.1186/1479-5876-10-83

**Published:** 2012-07-04

**Authors:** Paolo A Ascierto, Antonio M Grimaldi, Brendan Curti, Mark B Faries, Soldano Ferrone, Keith Flaherty, Bernard A Fox, Thomas F Gajewski, Jeffrey E Gershenwald, Helen Gogas, Kenneth Grossmann, Axel Hauschild, F Stephen Hodi, Richard Kefford, John M Kirkwood, Sancy Leachmann, Michele Maio, Richard Marais, Giuseppe Palmieri, Donald L Morton, Antoni Ribas, David F Stroncek, Rodney Stewart, Ena Wang, Nicola Mozzillo, Franco M Marincola

**Affiliations:** 1 Department of Melanoma, Sarcoma, and Head and Neck Disease, Istituto Nazionale Tumori Fondazione Pascale, Naples, Italy; 2 Earle A. Chiles Research Institute, Providence Cancer Center, Portland, OR, USA; 3 Department of Melanoma Research, John Wayne Cancer Institute at Saint John’s Health Center, Santa Monica, CA, USA; 4 University of Pittsburgh Cancer Institute, Pittsburgh, PA, USA; 5 Massachusetts General Hospital Cancer Center, Boston, MA, USA; 6 Laboratory of Molecular and Tumor Immunology, Robert W. Franz Cancer Research Center, Earle A. Chiles Research Institute, Providence Portland Medical Center, and Department of Molecular Microbiology and Immunology, Oregon Health and Science University, Portland, OR, USA; 7 University of Chicago, Chicago, IL, USA; 8 Department of Surgical Oncology, The University of Texas MD Anderson Cancer Center, Houston, TX, USA; 9 First Department of Medicine, Athens Medical School, University of Athens, Athens, Greece; 10 Huntsman Cancer Institute, University of Utah, Melanoma and Cutaneous Oncology Program, Salt Lake City, UT, USA; 11 Department of Dermatology, University of Kiel, Kiel, Germany; 12 Department of Medical Oncology, Dana-Farber Cancer Institute, Boston, MA, USA; 13 Westmead Institute for Cancer Research, Westmead Millennium Institute and Melanoma Institute Australia, University of Sydney, Sydney, NSW, Australia; 14 Division of Hematology/Oncology, Departments of Medicine, Dermatology, and Translational Science, University of Pittsburgh School of Medicine and Melanoma Program of the Pittsburgh Cancer Institute, Pittsburgh, PA, USA; 15 Medical Oncology and Immunotherapy, Department of Oncology, University Hospital of Siena, Istituto Toscano Tumori, Siena, Italy; 16 Molecular Oncology Group, The Paterson Institute for Cancer Research, Wilmslow Road, Manchester, M20 4BX, UK; 17 Unit of Cancer Genetics, Institute of Biomolecular Chemistry, National Research Council (CNR), Sassari, Italy; 18 UCLA Medical Center, Factor Bldg., 10833 Le Conte Ave., Los Angeles, CA, 11-934, USA; 19 Cell Processing Section, Department of Transfusion Medicine, Clinical Center, NIH, Bethesda, MD, USA; 20 Infectious Disease and Immunogenetics Section (IDIS), Department of Transfusion Medicine, Clinical Center and Center for Human Immunology (CHI), NIH, Bethesda, MD, USA; 21 Unit of Medical Oncology and Innovative Therapy, Istituto Nazionale per lo Studio e la Cura dei Tumori “Fondazione G. Pascale”, Via Mariano Semmola, 80131, Naples, Italy

## Abstract

After more than 30 years, landmark progress has been made in the treatment of cancer, and melanoma in particular, with the success of new molecules such as ipilimumab, vemurafenib and active specific immunization.

After the first congress in December 2010, the second edition of “Melanoma Research: a bridge from Naples to the World” meeting, organized by Paolo A. Ascierto (INT, Naples, Italy), Francesco M. Marincola (NIH, Bethesda, USA), and Nicola Mozzillo (INT, Naples, Italy) took place in Naples, on 5–6 December 2011. We have identified four new topics of discussion: Innovative Approaches in Prevention, Diagnosis and Surgical Treatment, New Pathways and Targets in Melanoma: An Update about Immunotherapy, and Combination Strategies.

This international congress gathered more than 30 international faculty members and was focused on recent advances in melanoma molecular biology, immunology and therapy, and created an interactive atmosphere which stimulated discussion of new approaches and strategies in the field of melanoma.

## Introduction

**Figure 1:**
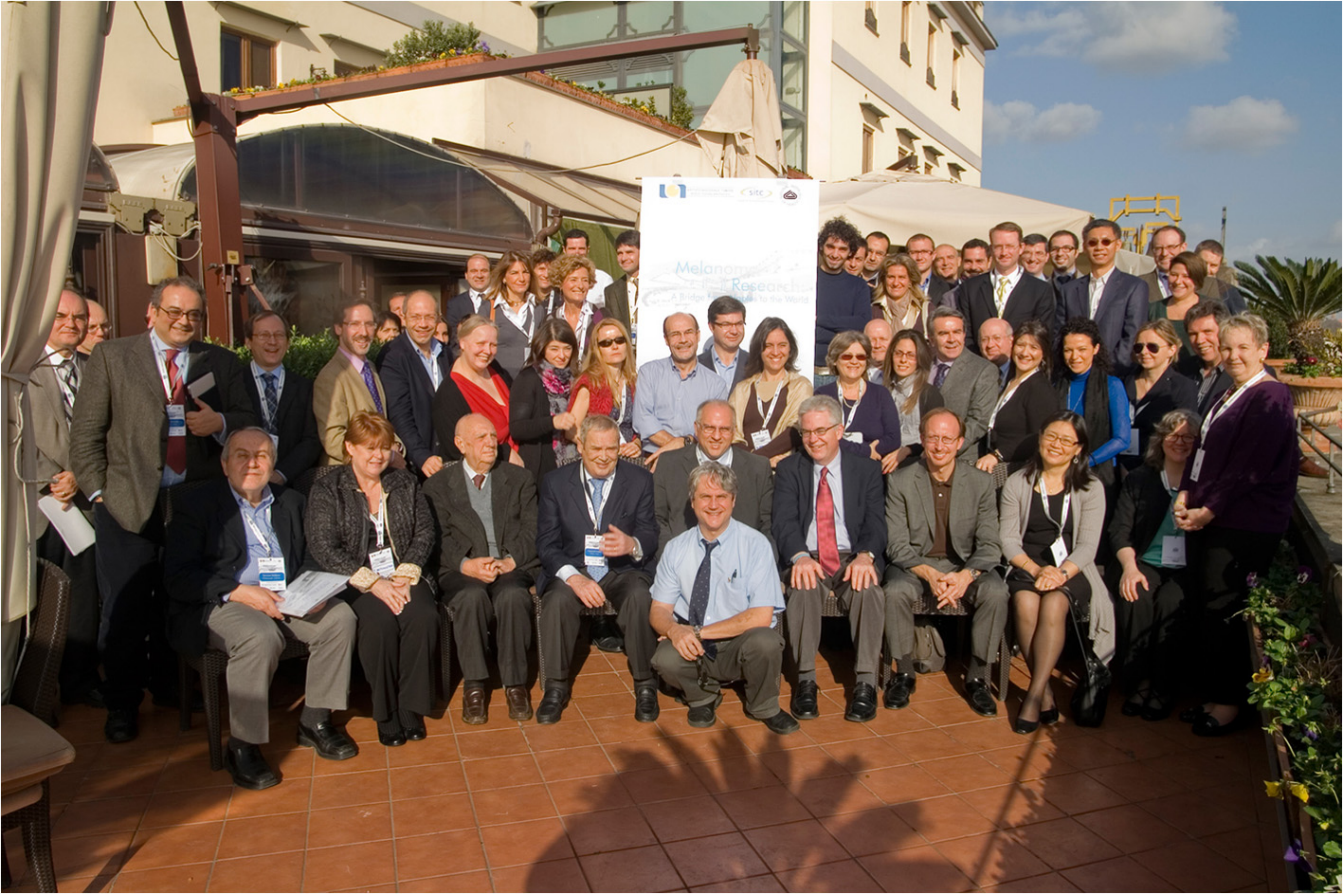
Majority of attendants of the Bridge meeting in Naples.

This year, the Melanoma Research Bridge meeting was held in Napoli on 5–6^th^ December 2011 (Figure 1). The scientific board selected four topics to be discussed during the two-day meeting: Innovative approaches in prevention, diagnosis and surgical treatment; New pathways and new targets in melanoma: an update; Immunotherapy: new evidence; Combination strategies.

The meeting started with a video lecture by Donald Morton about the role of surgery after the new active systemic medical therapy. Treatment of distant metastatic melanoma is still inadequate, as there were no systemic treatments with documented survival advantage until 2010/2011 with the approval of ipilimumab and vemurafenib. Prior to this, the 5-year median and overall survival for stage IV melanoma was only 8–10 months and 2.3%, respectively, while a meta-analysis by Korn et al. of all phase II cooperative group trials suggested that no systemic therapy evaluated in that setting was better than any other (that is, no better than no treatment at all). Ipilimumab, Antibody CTLA-4, was tested in two phase III trials and both showed a significant improvement in overall survival. However, grade 3 or 4 toxicity was reported in 56.3% of patients receiving ipilimumab, and the cost of the drug is over $120,000.

Vemurafenib, a selective BRAF inhibitor, demonstrated a survival benefit in one phase III trial (at 6 months OS was 84% vs. 64% for dacarbazine). However, only 50% of metastatic melanoma patients have the V600 BRAF mutation and most responses are transient (90% of patients progress within 9 months). New approaches to treatment of metastatic melanoma are still needed.

Conventional logic is that surgical resection (locoregional treatment) is not indicated with multiple metastases to distant organ sites because such patients have widely disseminated melanoma. But multiple series indicate long-term survival following resection of solitary distant metastases for melanoma, and a new look at surgery for metastatic melanoma is warranted. In fact 86% of patients presenting with distant melanoma metastases have only 1–3 sites of metastases in only 1 or 2 organs and only subsequently develop widespread disease. This suggests that there may be sequential progression or a metastatic cascade of disease from one site to another.

The rationale for cytoreduction in metastatic cancer is supported by: a) low morbidity and mortality (<1%) for metastasectomy; b) improved radiographic staging allowing for better selection; c) the observation that most patients have 1–3 initial sites of disease; d) the fact that the cost is less than many current medical therapies. Also, biologic evidence of the metastatic cascade derived from animal models suggests that metastases can metastasize. Clinical case reports support this evidence, and circulating tumor cell analyses have demonstrated a marked reduction of circulating tumor cells after resection of metastatic disease.

All of this leads to the hypothesis that one consideration for the initial treatment of metastatic melanomas is complete resection. Data supporting this hypothesis include those derived from several phase II trials of adjuvant immunotherapy after resection of stage IV melanoma. Several cases were presented featuring patients with multiple sites of disease resected over multiple years and then enjoying prolonged disease-free survival (8-13+ years). Sites of disease included lung, bowel, adrenal gland, and brain. Overall survival of patients with stage IV disease treated in this manner was 39% at 5 years and 30% at ten years.

Post-surgical adjuvant immunotherapy has also been pursued. A large randomized trial comparing a melanoma cell line-based vaccine with placebo (both groups received adjuvant BCG injections) in patients with resected stage IV melanoma was performed. Patients were stratified by M1a vs M1b/c and by the number of individual lesions (1, 2–3, 4–5). There was no difference in disease free survival (DFS) (HR 0.91 favoring vaccine, *p* = 0.418) or in overall survival (OS) (HR 1.18 favoring placebo *p* = 0.245). However, survival for both randomized groups was excellent (DFS: Median 8.3 mo vaccine, 7.2 mo placebo; DFS 5 yrs: 27.4% vaccine, 20.9% placebo; OS: Median 31.5 mo vaccine, 38.7 mo placebo; OS 5 yrs: 39.6% vaccine, 44.9% placebo). These excellent outcomes were seen for both M1a and M1b/c patients and there was no difference between patients with a solitary metastasis and those with 2–3 metastases. Even among patients with 4–5 metastases there were long-term survivors.

Among those patients whose disease recurs after initial metastasectomy, there is also a role for re-resection. From JWCI phase II data, 211 patients underwent initial metastasectomy. Among these patients 131 had recurrence and were managed non-operatively (n = 49), with complete resection (n = 40) or with incomplete resection (n = 42). Median survival (complete resection 17.4 months, incomplete resection 12.6 months, non-operative 5.6 months)(5 yr survival: complete resection 19.3%, incomplete 6.5%, non-operative 2.0%) suggest that patients may have benefitted from resection. Similarly in the MMAIT IV Canvaxin vaccine trial, among 308 patients with recurrence, 154 were treated surgically and 154 were treated without surgery. Median survival times (post-recurrence) were better for the surgery group (26.5 months vs. 9.0 months) as was the 5-year survival rate (26.5% vs. 7.0%).

Remarkably high survivals seen in phase II trials were confirmed in the phase III, multicenter trials for resection with adjuvant BCG. The source of these good outcomes is not clear and may be from patient selection or the effectiveness of surgery with or without BCG as an immune adjuvant. A new trial is underway to evaluate these possibilities. The trial enrolls patients with resectable stage IV melanoma and stratifies by sites of metastasis and number of metastatic lesions. Patients are assigned to one of three arms: surgery alone, surgery + BCG, and best medical therapy. Crossover is allowed at the time of progression and the primary endpoint is overall survival.

After Donald Morton’s lecture at the Bridge Congress a discussion began on the role of BCG as an adjunct to surgery alone. Among the topics discussed was the possibility that biology is driving the more favorable outcome of those patients, who are fully resectable for stage IV disease, in contrast to those patients who are not fully resectable and not considered for surgery because they have disseminated stage IV disease. In contrast to Donald Morton’s view, other oncologists argued that the difference in tumor biology (slow versus fast growing disease) accounted for the difference in survival and not the surgery. Published results of the two phase III trials on Canvaxin are expected shortly, but they will not provide evidence that surgery plus BCG alone is better than surgery plus BCG and Canvaxin. The results of surgery alone might be favorable, but can, at least in part, be explained by patient selection. The relative importance of surgical treatment versus tumor biology and patient selection remains controversial.

### Innovative approaches in prevention, diagnosis and surgical treatment

The meeting began with a discussion of the role about melanoma genetic testing in prevention and early detection. Melanoma-prone families comprise a minority of patients, but they have the greatest risk of developing the disease. Prevention and early detection play an essential role. To educate people on the risks of photodamage and melanoma, we must translate knowledge into changes in behavior; this means understanding cognitive processes. In a study at the Huntsmann Cancer Institute, the 52 patients enrolled were divided into three categories: p16 positive with a personal history of melanoma, p16 positive without a personal history of melanoma, and p16 negative without a history of melanoma. The study found that reporting of p16 genetic test results was associated with significant improvement in the frequency of performance of self skin examinations and a reduction in sunburns. Genetic test reporting also improved compliance with annual total body skin examinations by health care professionals in the p16 positive group that had not had a melanoma. Importantly, baseline compliance with these recommendations was poor when counseling was based on familial risk rather than on the genetic test report. These data suggest that the process of genetic test reporting enhances the patient’s ability to comply with prevention and early detection recommendations. The development of cognitive models that explain why genetic test reporting has this positive effect may lead to more generalizable and effective prevention education for sporadic melanoma as well. Larger trials are needed to further this effort.

Targetable chemoprevention pathways exist in melanoma and are being exploited in high risk patients. One pathogenetic mechanism for melanoma initiation is oxidative stress and resultant DNA damage while immune evasion is a mechanism in the promotion/progression phase. Based on the success of ASA in a high-risk human model (Lynch), a melanoma high-risk cohort is being recruited in preparation for analogous prevention trials in melanoma. Prevention has a greater potential impact than therapy on cancer because it impacts both morbidity & mortality and melanoma is an ideal cancer for prevention because it can be readily identified and has a well-established environmental cause. To summarize,, melanoma has targetable pathways that can be assayed in accessible tissues using relevant biomarkers in genetically characterized high-risk study participants.

A candidate chemoprevention agent for melanoma is sulforaphane. This agent has been isolated from broccoli sprouts (some studies have demonstrated that cruciferous vegetables are protective against cancer), and is an active agent identified by classic medicinal chemistry approaches with antioxidant activity. The antioxidant effect is accomplished via activation of the Nrf-2/ARE pathway and enhanced immunologic activity via STAT activation, leading to potential reversal of immuno-evasion. Predisposition pathways that are potentially targetable with sulforaphane include MC1R and p16. MC1R variants confer 2–4 fold increased risk for melanoma, while p16 mutation carriers have about a 76% lifetime risk for melanoma development. Importantly, p16 mutation carriers who also have an MC1R variant are at even higher risk. A novel oxidative stress function for p16 has been identified: RNAi knockdown of p16 leads to increased oxidative stress that can be reversed by an antioxidant and RNAi knockdown of p16 results in increased oxidative DNA damage. Genetic epidemiology studies also suggest that MC1R & p16 pathways may cooperate and this effect may be accomplished in part by simultaneously impacting oxidative stress pathways. Sulforaphane enhances antioxidant gene expression in melanocytes and in human epidermis ex-vivo and may effectively target oxidative stress, by bypassing the molecular defects in these high-risk groups. A Phase I/II chemoprevention trial of sulforaphane is needed to validate efficacy in surrogate nevi.

The Congress included an interesting discussion about the current concepts and future directions in melanoma staging and prognosis beyond the American Joint Committee on Cancer (AJCC)/ (International Union for the Control of Cancer (UICC) melanoma staging system. Overall, in the most recent version of the AJCC melanoma staging system (7^th^ Edition) no major changes were recommended for TNM and stage grouping criteria for stages I, II and III melanoma. Earlier models were validated using an evidence-based approach and an AJCC melanoma database comprising over 50,000 pts [1-3]. Highlights of revisions to the staging system include the following: (1) mitotic rate (measured in mitoses/mm^2^ using the dermal “hot spot” approach) was identified as independent prognostic factor, and based on a threshold of at least 1 mitosis/mm^2^, was included as a criterion for defining T1b melanoma; (2) immunohistochemical detection of nodal metastases is acceptable, and (3) there is no lower limit to designate N + disease [1-3]. These changes were also approved with Union for International Cancer Control (UICC) representation on the melanoma staging committee. In multivariate survival analyses in melanoma, mitotic rate was the second most powerful independent predictor of survival after tumor thickness [1,4]. Along with microstaging of all primary melanomas, pathological nodal staging for stage Ib-IIc melanoma helps to minimize prognostic heterogeneity within stages and incorporate sentinel lymph node assessment into the staging system [1,3].

Survival data of 7,635 patients with metastatic melanoma at distant sites (stage IV) sub-grouped by the site of metastatic disease and serum lactate dehydrogenase (LDH) levels were analyzed. As had been previously shown in earlier, albeit smaller studies, patients with distant metastatic disease only in the skin have a better survival than patients with lung metastasis or visceral metastasis; patients with lung metastasis also have a more favorable survival profile than patients with other visceral disease[1,3]. Importantly, patients with distant metastasis and elevated LDH levels also have a poorer survival than patients with normal LDH levels [1,3].

Limitations exist in traditional staging systems, and include the following: (1) number of characteristics that can be included - ie, patient, tumor, etc; (2) inability to use continuous variables; (3) estimates of survival based only on the time of diagnosis; and (4) TNM-based staging applies to large cohorts of patients, but is not truly individualized [5]. To improve melanoma staging and prognosis, it is evident that there is a need to develop and integrate new statistical models and contemporary analytic approaches that better inform using multiple characteristics and continuous variables, enhanced ability to combine evolving molecular features (eg, BRAF mutational status, PTEN expression) to better estimate cancer-specific survival in individual patient settings, and conditional probability models that estimate survival after treatment or at any time during follow-up [2,5-8]. Significant insight and clinical prognostic/predictive capacity driven principally by clinicopathological evidence-based risk-stratification are rapidly evolving. Tremendous strides in our understanding of the molecular underpinnings and heterogeneity of melanoma are beginning to enter current standard evaluation and management arena [9]. It is anticipated that identification of clinically relevant and “context-specific” biomarkers will facilitate staging and outcome predictions in patients with melanoma.

An update on Multicenter Sentinel Lymph node Trial (MSLT) Randomized Melanoma Trials was very interesting. MSLT 1 compared immediate versus delayed complete lymph node dissection for nodal metastases from melanoma >1.0 mm or ≥ Clark IV. Randomization (60:40) to either wide local excision with sentinel lymph node biopsy or wide local excision alone. Complete lymph node dissection was performed when nodal disease was diagnosed (either by SLN involvement or by clinical recurrence). Enrollment occurred from 1994–2002 and 2001 patients were enrolled. At the time of data lock (6/30/2011) 961 patients had completed 10 years of follow up, 672 died or have been lost to follow up and 210 remained on study. The current ongoing trial is MSLT 2, which examines whether complete lymph node dissection is necessary in the setting of a positive SLN. In most cases (approximately 88–89%) no additional metastases are discovered at the time of completion dissection. In addition the trial incorporates nodal ultrasound in follow up to facilitate early discovery of recurrence. In addition, those patients with involvement of non-sentinel nodes have very high systemic recurrence risks and may not benefit from additional prophylactic regional treatment. In MSLT2, patients with sentinel lymph node involvement (either by standard pathology or by multimarker RT-PCR) are stratified by Breslow thickness (>3.5 mm or <3.5 mm), site of sentinel lymph node (SLN) procedure (MSLT center or non-MSLT center) and degree of SLN involvement (pathology or RT-PCR) and randomized 1:1 to either completion lymph node dissection (CNLD) or observation with ultrasound and clinical examinations. Target accrual is 2000, and as of October 19, 2011 1,354 had been randomized. Enrollment is taking place at 63 sites around the world. Regarding the RT-PCR evaluation of samples from the trial, to date 1275 patients have had pathologically negative SLN screened by multimarker RT-PCR. Among these 1275 patients, 407 (24.2%) were positive of which 225 (55.3%) agreed to be randomized based on the PCR results and 188 (46.2%) accepted their randomization assignment. At the most recent meeting of the Data Safety Monitoring Board, it was concluded that an achievable sample size of 300 would not be adequate to determine if CLND was beneficial for RT-PCR positive patients. As such randomization based on RT-PCR was stopped. RT-PCR positive patients will continue to be followed for survival and prognostic information. The trial also evaluated ultrasound screening prior to SLN biopsy. As it is currently practiced around the world, ultrasound did not provide adequate sensitivity or specificity to be useful. This screening ultrasound has now been dropped from the trial.

After the presentation of the new data concerning the surgical treatment, the discussion focused on the current status of adjuvant treatment of melanoma patients and the possible selection of patients who might benefit. The aims of adjuvant therapy in high-risk melanoma are to reduce the risk of relapse, increase survival, provide treatment with tolerable safety profile. Interferon (IFN) is the only approved agent for the adjuvant therapy of melanoma. Patients may develop significant side effects frequently necessitating dose reduction or discontinuation of therapy. Mechanisms of action of IFN are to promote proliferation and clonal expansion of CD4 and CD8 T cells, to enhance antibody production of B cells, to increase cytotoxic activity of natural killer cells (NK) and CD8 T cells, and to have negative effects on the activation and proliferation of T regulatory cells (Tregs). Anti-tumor effects are anti-proliferative, anti-vascular, pro-apoptotic activity and modulating the immune response (role unclear). As showed by the meta-analysis of Mocellin, IFN benefits are analogous to other well established adjuvant treatments like in breast, colorectal and ovarian cancers, but no optimal IFN-α dose and/or treatment duration, or a subset of patients was identified to be more responsive to adjuvant therapy (4 months was the shorter duration of IFN administration). Molecular profiles may help in identifying patients who can benefit most from interferon adjuvant therapy. Most trials evaluating IFN used Breslow thickness and lymph node invasion for staging. This parameter was used for subgroup analyses of randomized control trials however the staging system was not identical over time. Subgroup analyses are hypothesis generating. Analyzing retrospectively the data of patients enrolled in two adjuvant EORTC trials, 18952 and 18991, the cohort of patients with ulcerated primary melanomas and microscopic lymph node involvement benefited in terms of replase free survival (RFS) and DMFS. This finding will now be prospectively validated in a EORTC trial which is enrolling patients with ulcerated melanomas.

In tissue studies performed in the context of a neoadjuvant trial, clinical responders had significantly greater increases in endotumoral CD11c + and CD3+ cells compared with non responders. In addition, HDI was found to up-regulate pSTAT1, whereas it down-regulates pSTAT3 and total STAT3 levels in both tumor cells and lymphocytes. Higher pSTAT1/pSTAT3 ratios in tumor cells pretreatment were associated with longer overall survival.

Pretreatment levels of proinflammatory cytokines (IL-1β, IL-1α, IL-6, TNF-α) were found to be significantly higher in the serum of patients with longer RFS values. Molecular HLA typing of patients receiving adjuvant IFN demonstrated that patients positive for HLA Cw*06 had a better relapse free and overall survival. These findings need to be prospectively validated in other adjuvant trials.

In 2013 the trial results of MAGE3 and Ipilimumab in the adjuvant setting will be available. MAGE-A3 is a tumor specific antigen. It is not expressed in normal cells, and it is therefore a good target for immunotherapy. It was identified via screening with anti-tumor killer T-cells. It is easy to detect in patients (RT-PCR on tumor tissue) and is present in major tumor types (lung, bladder, liver, melanoma) in early and advanced stages of a given disease and is potentially associated with poor survival prognosis. Based on the encouraging results of the phase II trial in metastatic melanoma, as well as the results of the phase II trial in adjuvant NSCLC and the high unmet medical need, a phase III trial was initiated in adjuvant melanoma. This phase III trial is called DERMA and has enrolled 1300 patients worldwide. To test Ipilimumab in the adjuvant setting two trials were designed: the EORTC trial of Ipilimumab vs placebo in stage III patients, that has completed accrual, and the ECOG 1609 study of Ipilimumab vs high dose interferon; the enrollment of this study started on May 2011. For patients with BRAF mutations some trials with BRAF inhibitors and/or combination with MEK inhibitors are currently underway.

Data were reported on electrochemotherapy (ECT), a new technology to treat melanoma patients. Electrochemotherapy is a combination therapy performed by electric pulses in association with a chemotherapic agent, generally bleomicin. The rationale underpinning this procedure is that external electrical stimulations can make cell membrane permeable to some molecules that in normal conditions cannot cross the membrane and penetrate into cells (electroporation). ECT is a method consisting of the combination of intra-tumoral injection of cytotoxic agents with the application of intensive electrical stimuli. Cliniporator is the device that permits the delivery of electrical pulses for this purpose. The electric pulses have high intensity (1000 V/cm), short duration (100μs), and can be repeated (8 pulses/nodule). When the electric pulses are applied to tumor cells, in 1500 ms, hydrophilic molecules normally excluded by the cell membrane, can enter inside the cytosol, by the formation of hydrophilic channels, and in 3 minutes, hydrophilic channels close and molecules migrate to nucleus. ECT allows drugs to reach the DNA and increase cytotoxicity (Bleomycin × 10,000 - Cisplatin × 80). ECT is performed by needles of different types and sizes for different indications (dimensions of the nodules, localizations of the nodules, etc).

In the ESOPE study, a phase II trial, electrochemotherapy, compared with bleomicin, was shown to be significantly more effective in metastatic tumour nodule treatment than the drug as single agent or electric pulses alone. Nodule complete response was confirmed by histological and immunohistochemistry analysis. Higher response rates were obtained in melanoma nodules. At the National Cancer Institute in Naples tumor nodules from 86 patients with different diagnosis were treated with ECT: 38 patients with melanoma, 18 with basal cell carcinoma, 12 with Kaposi’s Sarcoma, 9 with squamous cell carcinoma, 5 with breast cancer, 2 with pancreatic cancer and 2 with bone metastasis. A total of 126 ECT treatments were performed, distributed as follows: in 38 patients with melanoma nodules, one or more treatments; in 16 patients with basal cell carcinoma, two or more treatments; in 7 patients with Kaposi’s Sarcoma, three or more treatments; and in 3 patients with squamous cell carcinoma, four or more treatments. ECT can be curative, if it results in the disappearance of treated nodules; palliative, if it stables disease and reduces pain; hemostatic, if it stops bleeding, or neoadjuvant, if it reduces the size of the disease that can then be surgically removed. The most common side effects of ECT are erythema, electrodes tattoo, erosion or ulceration with scaring, slight oedema and pain.

ECT is a simple, safe, economic, highly effective and cosmetic repeatable procedure with a short learning phase, that improves the quality of life independent of life expectancy.

### New pathways and new targets in melanoma: an update

DNA methylation is known to control gene expression of multiple pathways relevant to melanoma. Examples of specific changes include hypermethylation of CDKN2A, MGMT, and PTEN, and hypomethylation of key antigens such as the Melanoma Antigen family (MAGE) loci and NY-ESO-1. While methylation of promoters is governed by DNA methyltransferases (DNMTs) the factors responsible for demethylating DNA have only recently been identified. Active demethylation has long been suspected based on evidence such as the IL-2 promoter’s demethylation within 20 minutes after stimulation of naïve T-cells in vitro. Recent work at the Huntsman Cancer Institute has shown that a trio of proteins including activation induced deaminase (AID), Gadd-45, and MBD-4 work in concert to demethylate DNA in zebrafish embryos. These factors may drive some of the abnormal methylation patterns seen in melanoma, and may maintain cells in a more stem-cell like state. In efforts to improve the therapeutic effectiveness of immune therapy, drugs targeting the DNMTs have shown successful re-expression of melanoma antigens *in-vitro* and in patients, and have improved response rates to IL-2 therapy. Limitations of currently available epigenetic modifiers include relatively short half-lives, and concominant DNA damage leading to cytopenias. In efforts to circumvent these problems, new di-nucleotide-based compounds designed at Supergen have shown greater stability than previous demethylating agents such as 5-Aza deoxycytidine and show favorable pre-clinical toxicity profiles. As future studies directed towards improving response rates in immunotherapy, and circumventing drug resistance occurring with targeted therapy will likely employ epigenetic modifiers, more stable compounds such as these may be more desirable for combination studies in melanoma.

Clinical and pre-clinical studies with molecular targeted therapy reveals a dependence on MAPK signaling for melanoma tumor growth and maintenance, and re-activation of the MAPK pathway through direct and parallel pathways appears to be essential for mediating drug resistance and tumor progression [10]. During neural crest development the MAPK pathway controls a highly conserved transcriptional response that involves repression of FOXD3 mRNA and protein, which in turn activates MITF expression to promote melanocyte migration and differentiation [11-13]. This response remains intact in melanoma cells, as inhibition of the MAPK pathway causes re-expression of FOXD3, which in turn causes cell cycle arrest, increased cell survival, decreased migration, loss of differentiation markers (pigment); properties consistent with a transient progenitor state [14-16]. Indeed, MAPK-inhibited melanoma cells express higher levels of neural crest progenitor/stem cell markers such as *DCT* and *SOX10*. These studies suggest that inhibition of the MAPK pathway causes a subset of melanoma cells to de-differentiate into a multipotent cell population, which is more resistant to cytotoxic apoptosis. Future *in vivo* studies will be needed to determine the consequence of FOXD3 re-expression in melanoma cells after BRAF-inhibitor treatment to determine 1) if FOXD3 is a useful biomarker for drug-dependent tumor regression and 2) if melanoma cells de-differentiate. If BRAF-inhibitor induced de-differentiate occurs, our knowledge of the embryonic neural crest pathways that control melanocyte development can be exploited to identify synthetic lethal interactions that rely on FOXD3 re-expression and its role in differentiation of other neural crest lineages, such as glia, eg., biological therapies.

ERK has a pivotal role in melanoma because this pathway is hyperactivated through gain-of-function mutations in the majority of melanoma cases. Primarily, this is driven by mutations in genes such as *BRAF* (44% of cases), *NRAS* (22%), *ERBB4* and *cKIT* (5% each). Some of these driver oncogenes are validated therapeutic targets and in randomized clinical trials, BRAF inhibitors can mediate extraordinary responses [improved progression free survival (PFS) and median OS], in patients with V600BRAF mutations. Curiously however, a frequent side effect of BRAF inhibitors is the induction of cutaneous squamous cell carcinomas (cuSCC), which is driven by a paradoxical activation of the MAPK pathway in pre-cancerous keratinocytes that carry oncogenic mutations in RAS genes. Surprisingly, nilotinib, a selective Bcr/Abl tyrosine kinase inhibitor, also drives paradoxical RAF activation and synergizes with MEK inhibitors to kill drug-resistant CML cells. These data highlight the importance of understanding the genetic landscape of individual tumours and emphasize the potential of complete genome sequencing to provide better understanding of human cancer.

The AMP activated protein kinase (AMPK) controls energy homeostasis in cells by measuring AMP/ATP ratios. In metabolic stress, AMPK restores energy balance by increasing energy production and blocking energy consuming. Intriguingly, whereas most cancer cells are sensitive to the growth inhibitory effects of AMPK activation, BRAF mutant melanoma cells are resistant to AMPK through the action of the protein kinase RSK. Furthermore, in vivo, AMPK activators drive the production of VEGF-A in BRAF mutant melanoma cells and the combination of metformin and VEGF signaling inhibitors drive a synthetic interaction that blocks the growth of BRAF mutant melanoma cells in vivo.

BRAF mutations are expressed in about 50% cutaneous melanomas (20% continuous sun-exposure, 50–80% intermittent sun-exposure), and in areas of high sun exposure, like Australia, 80% mutations are V600E, and this kind of mutation is present in about 90% of patients between 20 and 40 years-old. Vemurafenib and dabrafenib are two powerful BRAF inhibitors that give a high response rate in a very short time in BRAFV600 mutated melanoma patients and have good activity even in brain metastases. About 50% of mutated patients respond to BRAF inhibitors. In the BRIM 3 trial vemurafenib had a PFS or 5.3 months, and in the phase II BRIM 2 trial PFS was 6.7 months; the BREAK-2 trial of dabrafenib showed even different PFS in patients with V600E mutation and V600K, with an advantage for “E” mutation (27.4 vs 19.7 weeks). The BRIM 3 trial showed an important advantage even in overall survival with 83% 6 month survival for vemurafenib vs 63% 6 months survival for dacarbazine. However, patients tend to relapse; about 5 patterns of relapse have been described, but generally (43%), the progression is in new sites only, while in 21% it is in pre-existing site only.

To continue BRAFi treatment beyond progressive disease (PD) resulted in good outcomes in anecdotal reports; prolonging therapy *beyond* PD could mean prolong survival (median survival beyond PD >9 vs 3 mths *p* = 0.008), but this type of strategy calls for a randomised discontinuation trial. This effect may be due to a “tumour flare” on BRAFi withdrawal, even after PD.

MEK inhibitors as single agents have activity against mutated BRAF melanoma, unexposed to prior BRAF inhibitor therapy, but they won’t salvage BRAF inhibitor resistance. A new combination of the MEK and BRAF inhibitors trametinib and dabrafenib as first line therapy for BRAF mutated melanoma patients is showing great promise. In BRAF^V600E^ human melanoma xenograft BRAFi + MEKi showed enhanced antitumor activity, with more sustained tumor control than that seen either single agent. This combination of BRAF and MEK inhibitors is obtaining very good results in melanoma patients naïve to prior anti-BRAF treatment, with about 5 complete responses, and a high tumor reduction rate. 83% of these 77 patients were ongoing at 30 weeks of treatment, when the study was presented. However, even this combination needs to be evaluated in new randomized clinical trials.

Resistance to BRAF inhibitors is mediated by different mechanisms as shown from about 60% of biopsies performed in progressing lesions. Among these mechanisms the most reproducible in patient-derived samples are secondary NRAS mutations, upregulation of RTKs (PDGFRβ, IGF1R) and BRAF truncations. The mechanism of resistance may predict for sensitivity to the addition of secondary treatments such as growth factor receptor inhibitors or PI3K/AKT/mTOR inhibitors. Combining immunotherapy and BRAF targeted therapy is possible; vemurafenib does not adversely affect the function of human or murine lymphocytes; the combination of vemurafenib with anti-CTLA4 immunotherapy is mediated by improved intratumoral infiltration by activated lymphocytes in a fully syngeneic and immunocompetent mouse model of BRAF^V600E^ mutant melanoma; a phase 1 clinical trial of a combination of vemurafenib and ipilimumab is ongoing.

### Immunotherapy: new evidence

The development of the first tumor antigen-specific monoclonal antibodies dates back to the '70s. The characteristics of these reagents in terms of specificity, reproducibility and availability in large amounts generated a lot of hopes and enthusiasm about the clinical application of immunotherapy for the treatment of malignant diseases. Unexpectedly most if not all the clinical trials yielded negative results. As a result the scientific community became skeptical about the clinical usefulness of tumor antigen-specific monoclonal antibodies to develop immunotherapeutic strategies for the treatment of malignant diseases. Things changed in 1997 when rituximab and trastuzumab were approved by FDA for the treatment of non Hodgkin lymphoma and breast cancer, respectively. In the following years a growing number of tumor antigen-specific monoclonal antibodies have been approved and several of them have become part of the therapeutic armamentarium used for the treatment of malignant diseases.

Among the many tumor antigens which are being evaluated as potential targets of immunotherapy, the membrane bound chondroitin sulphate protidoglycan 4 (CSPG4), which was initially named High Molecula Weight-Melanoma Associated Antigen, certainly deserves mention. This target is expressed with high density on the cell membrane of many types of malignant cells. They include melanoma (~85%), glioma (~70%), triple negative breast cancer (~50%), mesothelioma (~50) chordoma and chondrosarcoma (~50%), and acute lymphoblastic leukemic (~55%) lesions. Furthermore CSPG4 is upregulated on activated pericytes in the tumor microenvironment; as a result, CSPG4 immunotargeting may inhibit neoangiogenesis in the tumor microenvironment and suppress growth of tumor cells, even if they do not express CSPG4. In view of the postulated role played by cancer initiating cells in metastatic spread and in disease recurrence it is noteworthy that CSPG4 is expressed on cancer initiating cells at least in melanoma, head and neck cancer and breast cancer. Because of the interest in utilizing CSPG4 as a target of immunotherapy, it is noteworthy that this antigen has a restricted distribution in normal tissues. CSPG4-specific mAb have been found to be effective in inhibiting the growth of human melanoma cells and their metastatic spread in immunodeficient mice. This effect is mediated by the inhibition of several signaling pathways including the ERK and FAK pathways.

Another potential target of antibody-based immunotherapy discussed at the meeting is glucose regulated protein of 94,000 daltons (Grp94). Grp94, a member of the Heat shock protein (HSP) 90 family, is located in the endoplasmic reticulum of all mammalian cells. This chaperone is essential for the conformational maturation of several proteins that play key roles in transducing proliferative and anti-apoptotic signals. These functional properties of members of the HSP90 family have provided the rationale for the clinical use of HSP90 inhibitors for the treatment of malignant diseases with the expectation that the inhibition of its chaperone function induces the degradation of its “client” proteins. Therapeutic effects have been observed. However the clinical use of these inhibitors is hampered by the associated side effects. These clinical findings emphasize the need to develop strategies to overcome the limitations. In this light the fully human mAb W9, which was described at this meeting, is of great interest, since it recognizes an extracellular epitope of Grp94. This epitope is selectively expressed on malignant cells. mAb W9 inhibits the proliferation of tumor cells; this effect is mediated by the inhibition of several signaling pathways.

Ipilimumab improves survival in previously treated metastatic melanoma patients (+/− CNS disease) compared to gp100 peptide vaccine (HR = 0.68), and in association with dacarbazine improves survival in untreated patients with metastatic melanoma compared to dacarbazine alone (HR = 0.72), with 10% high grade adverse events. To improve on these results clinical investigators are testing different strategies of therapy such as integrating cancer vaccines and CTLA-4 antibody blockade. Concurrent therapy with GM-CSF-based vaccines in murine tumor models have revealed potent therapeutic synergies, but associated with toxicity; moreover CTLA-4 enhances immunologic memory responses. GVAX (granulocyte-macrophage colony-stimulating factor [GM-CSF] gene transduced irradiated melanoma vaccine cells) offers the possibility that “host versus melanoma” immune responses can be generated in melanoma patients. At the Dana-Farber Cancer Institute, a trial of anti-Ab CTLA-4 enrolled 14 stage IV melanoma patients pretreated with GVAX, and treated them with 3 mg/kg ipilimumab every 2–3 months. In the 14 GVAX patients, this combination obtained three partial responses, one partial response (near CR) following DTIC and six stable disease with a median duration of 30 months. Possible Mechanisms of action of GM-CSF-based vaccination + CTLA-4 blockade can be the expansion of primed anti-tumor immune effector cells; this association allows CTLA-4 blockade to selectively target anti-tumor effector cells (i.e. therapeutic index). In attempts to simplify the therapeutic strategy of combining GM-CSF biology with immune checkpoint blockade, the Eastern Cooperative Oncology Group planned a Phase II Trial of GM-CSF Protein Plus Ipilimumab in Patients with Advanced Melanoma randomizing melanoma patients to receive Ipilimumab 10 mg/kg induction/maintenance plus GM-CSF 250 μg days 1–14 in a 21 day cycle or Ipilimumab alone. The primary endpoint is overall survival.

Humoral responses to VEGF and angiopoietins have been associated with clinical benefit in some patients receiving therapeutic vaccines. Importantly, VEGF has known immune modulatory effects, specifically decreasing dendritic cell maturation. Basing on these considerations,started a phase I clinical trial with Ipilimumab plus bevacizumab. Melanoma patients were first treated in two cohorts, one treated with 10 mg/kg ipilimumab plus 7.5 mg/kg bevacizumab and another with 10 mg/kg ipilimumab plus 15 mg/kg bevacizumab, with induction of ipilimumab every 3 weeks × 4 cycles then every 3 months maintenance, and a maintenance with Bevacizumab continued every 3 weeks. Of 22 patients treated to date, clinical activity has been observed. CTLA-4 plus VEGF-A blockade may have effects on both tumor immunity and tumor vasculature. Randomized phase II and III trials will be needed to discern the effect of the addition of VEGF-A blockade to CLTLA-4 blockade.

Features of the tumor microenvironment could dominate at the effector phase of the anti-tumor T cell response and limit efficacy of current immunotherapies. Systematic analysis of the tumor microenvironment could identify a predictive biomarker profile associated with clinical response, and also highlight new biologic barriers that need to be overcome to optimize therapeutic efficacy of vaccines and other immunotherapies. An “inflamed” gene expression pattern of tumor microenvironment has been associated with favorable clinical outcome to multiple vaccine platforms in melanoma. Ipilimumab clinical responders also appear to show an “inflamed” tumor gene expression profile. Therefore, an inflammatory gene expression profile in metastatic melanoma might have utility as a predictive biomarker for response to vaccines and other immunotherapies. Post-vaccination, increased CD8 transcripts combined with decreased melanoma antigen transcripts in the tumor is a pattern associated with clinical benefit. One major barrier to effective immune-mediated tumor destruction is poor T cell migration and the “non-inflamed” subset of patients. Still, T cell migration into tumors appears to be necessary but not sufficient for clinical response. Inflamed melanomas containing CD8^±^ T cells have highest expression of immune inhibitory pathways including IDO (indoleamine-2,3-dioxygenase)-induced tryptophan catabolism, PD-L1 engagement of PD-1 on T cells, extrinsic suppression by CD4^+^CD25^+^FoxP3^+^Tregs and T cell anergy due to poor expression of B7 costimulatory ligands. The underlying mechanism explaining “inflamed’ versus “non-inflamed” tumor microenvironment are not yet understood. Possibilities being explored include inter-patient heterogeneity at the level of oncogene pathway permutations within the tumor cells, germline polymorphisms at the level of the host, or differences in gut flora commensal organisms, “Inflamed” tumors likely are not rejected due to dominant immune suppressive mechanisms (IDO, PD-L1, Tregs, Anergy), which are all potential therapeutic targets. Increased PD-L1, IDO and Tregs in the tumor site are driven by CD8^+^ T cells in the tumor microenvironment. Blockade of these pathways is being explored in the clinic, already with preliminary progress. A new set of surface markers driven by EGR2 may provide a strategy for identifying intrinsically dysfunctional CD8^+^ T cells from the tumor microenvironment and LAG3 and CRTAM are candidate therapeutic targets.

Melanoma is definitely not a status quo, but an evolving process included as part of an intracellular network of interconnections, influenced by several factors such as the genetic basis of the individual subject, the genetics make up of the disease and environmental factors. To understand the immune mediated tumor rejection, a holistic approach that capture the complexity entity of the given time and condition instead of focusing on single or limited parameters should be considered, especially when the mechanism is elusive. Transcriptome analysis of the tumor microenvironment under a variety of immunotherapies has uncovered a common gene expression pattern represented by activation of key immune modulators such as IRF1, START1, T-bet, IFNG and IL15; up regulation of effector molecules such as GNLY, GZM and TIA accompanied by over expression of CXCR3 and CCR5 with corresponding ligands (CXCL9-11, CCL5). The impact of this same gene signature on the response to anti-tumor immunotherapy are indicative of immune mediated tissue destruction such as in autoimmune disorders, acute infection clearance and transplant rejection suggesting a converging mechanism independent of the causal initiation. It is even more conceivable that this same gene signature with consequent changes in the level of transcription in tumors is increasingly important as a biomarker associated with good prognosis and survival. Gene sets found to be highly correlated with clinical response are the Interferon-Gamma-pathway, AKT pathway, CCR5 pathway and NKT pathway. Majority of effector function related genes are down-regulated while proliferation and cell cycle related genes are up regulated suggesting a phenotypic defined immune cell subset in CR different from NR which may be responsible for the potent effector function and possible mechanism of rejection. A prediction model developed based on those significant genes can accurately predict about 75% of melanoma patients clinical outcome under adoptive TIL therapy, although, those data need to be validated in an independent study. However, the down-regulated genes could be result of the intrinsic genetics heterogenity (CCR5, CXCR3 and IRF5) of the patient which has intrinsic impact to the tumor.

Genetic polymorphism, the essence of human heterogeneity, play an important role in diverse disease susceptibility and impact the natural history of disease. Polymorphism of IRF-5 appears to be a predictor of immune responsiveness of melanoma metastases to adoptive therapy with TIL. The rs10954213 G allele, which is protective against SLE, is the most predictive of non responsiveness suggesting a correlation between autoimmunity and melanoma immune responsiveness. The expression profile of TIL classified according to AA vs GG IRF5 rs10954213 (G > A) appears to be a borderline predictor of immune responsiveness. The expression profile of pre-treatment melanoma metastases classified according to AA vs GG IRF5 rs10954213 (G > A) appears to be a stronger predictor of immune responsiveness compared with TILs suggesting possible involvement of tumor microenvironment. However, comparison of melanoma cell lines derived from the pretreatment melanoma lesions classified according to the AA vs GG IRF5 rs10954213 (G > A) highlights a signature of genes that differentiates the two genotypes clarified that the genotype of the tumor cells itself make the difference independent of micro environmental influences. The signatures differentiating the two cell line genotypes in vitro could predict of the responsiveness of melanoma metastases in vivo suggesting that immune responsiveness is at least in part genetically determined. Thus, it appears that immune responsiveness is at least in part dependent on the genetic background of the host which affects the biology of cancer cells primarily and secondarily the immune responsiveness of tumors.

The major challenge for the field is how to monitor the antitumor immune response for non-antigen-specific immunotherapy such as anti-CTLA4, anti-PD1 and IL-2 and for antigen-specific immunotherapy since the fact that the antigen is administered, doesn’t mean that immune system “sees” only that specific antigen (epitope spreading). We do not know which parameters of immune responses and which assays used to assess these parameters are optimal for efficacy analysis. There is a need for the development and validation of tools to identify patients who can benefit from a particular form of immunotherapy. The analysis of single parameters alone may not provide sufficient insights about complex immune system–tumor interactions. Common immunoassays do not take into account changes in the differentiation of immune cells, in the antigenic profile of tumors and responding T cells, in T-cell homing receptors, or the complex analysis of responses to “private” antigens or epitope spreading. The development of protein arrays that contain 9000 human proteins are being used to identify the generation of antibody responses following immunotherapy. Since production of IgG antibody responses require CD4 help, identification of a new or increased IgG antibody response following immunotherapy potentially provides a surrogate for generation of an anti-tumor T cell response. This strategy is being employed by several groups to characterize the immune response following immunotherapy and holds promise as a strategy to monitor responses against a wide range of possible targets.

Tumor infiltrating lymphocyte (TIL) therapy has been the cornerstone of adoptive cellular therapy of melanoma. TIL therapy is changing and other adoptive cell therapies are now available [17]. Recent improvements in TIL therapy of melanoma include the use of lymphodepletion recipient preparative regimens and more rapid TIL production – young TIL [18]. The beneficial effects of leukocyte depletion are likely due to the elimination of Tregs and increased serum cytokine levels that result in greater in vivo TIL persistence and expansion which have resulted improved clinical outcomes [19]. The in vivo persistence of young TIL is greater than classical TIL, but the clinical benefits of young TIL therapy are still being evaluated.

When TIL therapy is not possible because metastatic tumor can’t be resected or TIL can’t be isolated from resected tumor, genetically engineered autologous T cells can be used for adoptive T cell therapy. Autologous T cells that were been genetically engineered to express a high affinity T cell receptor (TCR) specific for the cancer testis antigen NY-ESO-1 have used to treat melanoma and sarcoma [20]. Preliminary results of adoptive cell therapy using T cells with genetically engineered TCRs have been promising but TCRs are HLA-restricted, the required vectors are expensive and gene transduction is technical difficult. In the future, the use of autologous naïve and stem cell-like memory T cells may further enhance adoptive cell therapy using genetically engineered T cells [21].

Culturing and expanding TIL for clinical therapy is technically demanding, expensive and time consuming which has restricted the clinical use of this therapy. Recently, it has been found that TIL production can be improved by using gas permeable G-Rex flasks for initial TIL culture and rapid expansion [22]. The benefits of this method of TIL production are lower final volume and fewer flasks and no electronic or mechanical devices are required.

### Combination strategies

The rationale for adjuvant therapy lies in the greater responsivness of micrometastatic and operable regional disease (Stage IIIA-B), as compared to inoperable advanced (stage IV) disease. Adjuvant therapy with IFN reduces the hazard of relapse and mortality by 33%, whereas multiple studies have shown response rates in advanced stage IV disease that are in the range of ~16% [23]. The presence of advanced inoperable disease has immunomodulatory consequences that have been documented by Tatsumi and Storkus [24]. The objective response rates observed with immunotherapies beginning with IFNα have been to be inversely correlated with the disease burden. The trials E1684, E1690, and E1694 show how durable and significant the impact of IFN upon relapse-free and overall survival. Three meta-analyses of the aggregate of all trials that have been conducted with IFNα confirm RFS and OS benefits of IFN [25-27]. However, it has not yet been estabilished what the optimal dose, route, and duration of IFN therapy are. All trials conducted with IFNα show unequivocal and durable benefits in terms of RFS but only two independent trials have shown both RFS and OS impact, both of which utilized IV induction at 20MU/m^2^ followed by SC maintenance IFN at 10MU/m2 for a full year of treatment.

Two trials, the Intergroup E1697 and Neoadjuvant Trial UPCI 00–008 have tested the effects of one month of IV IFNα2b. The phase III intergroup trial E1697 compared 1 month of iv high-dose IFN vs. observation, demonstrated the lack of durable benefit of the 1 month treatment in mature data released in in stage IIB/IIIA resected melanoma patients with futility analysis at 1155 patients [28]. The neoadjuvant trial UPCI 00–008 conducted in patients with bulky lymph node metastatic disease showed significant antitumor effects in 55% of patients with stage IIIB-C disease assessed at 1 month, as well as significant immunomodulatory effects in patients receiving the 1 month iv high-dose regimen—so we conclude that the one month regimen is active, but that durable benefits of this agent require longer than 1 month of administration. The search for biomarkers that correlate with antitumor benefits of IFN has been a critical undertaking. Patients with the development of serological or clinical signs of autoimmunity during HD-IFN derive the greatest benefit in terms of PFS and OS [29]. But the serum cytokine/chemokine profile can predict treatment benefit with HDI: in fact, baseline pro-inflammatory cytokine levels were found to predict 5-year relapse-free survival in patients treated with High-Dose IFNα. The updated data from the EORTC 18991 trial showed benefit from this 5 year Peg-IFN regimen that diminished at 7.6 years, compared with the earlier published analysis and there is no significant impact upon DMFS or OS either early or at 7.6 years maturity in this trial. Analyzing the subgroup of with stage III N1 disease shows significant RFS and DMFS impact in 2007, but at 7.6 years this is no longer statistically significant; patients with stage III N2 showed no benefit in any of the several endpoints, and patients with primary tumor ulceration analyzed at the 7.6 year time point show the greatest benefit of Peg IFN among the subset of patients with Stage III N1 disease and ulcerated primary tumors (median OS of peg IFN vs. observation: > 9 vs. 3.7 years).

New adjuvant strategies have been tested more recently, but among mature phase III trials only HDI demonstrates confirmed significant durable OS & RFS benefit at >20 years (E1684/90/94). A variety of tumor cell vaccines have been assessed giving largely disappointing results: Canvaxin was shown to be ineffective and possibly detrimental in Ph III trials for both stage III and IV resectable tumor; GMK, a ganglioside GM2 vaccine administered with QS21 adjuvant conjugated to the KLH carrier, was inactive and MAGE A-3 results are pending. Neither GMCSF nor peptide vaccination improved OS or DFS overall in the ECOG led intergroup US study E4697 (the trends to benefit among Stage IV subjects will require further study), and Anti-CTLA4 blocking mAbs (tested in EORTC trial 18071, and US Intergroup trial E1609) will not mature for some time. BRAF and MEK inhibitors are planned for evaluation but these studies are not yet launched.

Ipilimumab has been studied by Medarex-BMS in the 020 and 024 trials, each demonstrating significant durable benefits in advanced unresectable patients with metastatic melanoma—so the evaluation of this agent in the adjuvant setting is reasonable, as already discussed: the larger question that remains unanswered is which dosage of ipilimumab will be most effective—as the FDA has approved the dosage of 3 mg/kg but the EORTC 18071 trial has only evaluated the dosage of 10 mg/kg, compared to placebo. The US Intergroup trial E1609 has addressed this with recent modifications that will evaluate both 10 mg/kg and 3 mg/kg vs the active standard of HDI.

The neoadjuvant setting has already been alluded to, as it may give rapid and mechanistic answers regarding new potential adjuvant therapies. Neoadjuvant High-Dose IFN-α2b was studied in the trial UPCI 00–008 that showed clinical responses at day 29 in 55% of patients, and a molecular impact upon STAT3 with reduction of the pSTAT3/STAT3 constitutively expressed in tumor tissue. This study also showed modulation of IFNAR2 and increased expression of pSTAT1, and TAP2 in tumor tissue. The immunologic impact upon CD3 T cell, and DC responses to tumor (with increased CD3 T cell and CD11c dendritic cell populations in tumor) provided the strongest evidence of the immunomodulatory mechanism of IFN adjuvant therapy. Neoadjuvant therapy with Ipilimumab at 10 mg/kg has now been tested as presented by A. Tarhini, [30]. These interesting results mirror results obtained with tremelimumab + HDI that have recently been published in advanced melanoma [31]. A current neoadjuvant trial of Ipilimumab 10 mg/kg or 3 mg/kg + HDI will also shed light on dose–response effects of ipilimumab at the two different dosages, combined with high-dose IFN.

The effects of immunotherapy in melanoma are observed in the tail of the survival curves, with long term survivors, while the major effects of targeted therapy for melanoma occur in the initial splay of the curve with high response rates. In patients with metastatic melanoma harboring BRAF V600 mutation, vemurafenib has achieved striking results in terms of PFS and OS. This agent has yet to be evaluated in the adjuvant setting, but its effects in relation to tumor debulking, increased T cell infiltrates in some series, and possibly increased antigenicity and APC function may translate to improved adjuvant therapeutic benefits: however, the finite durability of benefits, and the absence of mature survival data in phase III trials qualify this assessment. It may be that BRAF inhibitors are most useful as partners in combination with IFN for the adjuvant therapy of bulky disease, to capitalize upon immunomodulatory functions of BRAF inhibitors, and to limit the necessary interval of BRAF inhibitor therapy. Phase II data are needed for IFN-BRAF combinations and this will be one area for future exploration.

Adjuvant application of molecularly targeted therapy in combination with immunomodulators offers opportunity to magnify therapeutic impact of the immunotherapies, and to obtain more durable benefits from the molecularly targeted therapies. Whether agents that do not induce durable CR or durable disease control in stage IV will have benefits in the adjuvant arena is now testable.

In 2008, Korn performed a meta-analysis of phase II cooperative group trials in metastatic stage IV melanoma aimed at determining progression-free and overall survival benchmarks for future phase II trials [32]. The results were daunting, since only 25.5% of the patients treated in these phase II studies were alive at 1 year (median PFS, 1.7 months; median OS, 6.2 months). From that time, history has however changed in regard to two new modalities, due to the approval and the introduction into the clinics of innovative new drugs. Until 2010, just two chemotherapeutic agents were available for the treatment of metastatic melanoma: Dacarbazine and (in Europe) Fotemustine and (in the US) Aldesleukin. In 2011, Ipilimumab was approved for both first and second lines in USA or solely for second line in Europe and Vemurafenib was approved for first and second lines in ^V600E^BRAF mutated patients. Both the drugs gave effective but different results (i.e. Ipilimumab, compared with Dacarbazine, provides an important advantage in OS but not in PFS, while Vemurafenib impacts on both PFS and median OS), reflecting different mechanisms of action and kinetics [33-35].

In this regard, new strategies for the therapy of melanoma have used the combination of different drugs with different mechanisms of action. Some examples of ongoing trials are: a dose-escalation study of the combination of anti-PD1 and Ipilimumab (NCT01024231) in subjects with unresectable or metastatic melanoma; a study of RO5185426 and GDC-0973 in patients with BRAF-mutation positive metastatic melanoma; and a phase I/II Ipilimumab Vemurafenib combination (NCT01400451). A fundamental differentiation for prognosis and, above all, therapeutic effects is the distinction of all patients in two main subgroups: BRAF-mutated and BRAF-wild-type. In patients with ^V600E^BRAF mutation and, thus, oncogenic activation of the MAPK pathway, targets that can be hit are BRAF, MEK, and, probably, ERK. Selective BRAF inhibitors are Vemurafenib and Dabrafenib. Both of them, compared with Dacarbazine, obtained an advantage in response rates, PFS and OS; however, a new BRAF inhibitor is now under evaluation, LGX818 (Novartis), and new therapeutic strategies are ongoing in clinical trial, such as Vemurafenib + Surgery or Radiotherapy in patients presenting progression during therapy with Vemurafenib. At 2011 ASCO Meeting, Kim showed how the treatment beyond progression with Vemurafenib does impact on OS among BRAF-mutated patients [36]. Another therapeutic target is MEK: there are at least five MEK-selective inhibitors, and GSK1120212 (GSK) has been demonstrated to achieve better results in BRAF-mutated patients non pre-treated with BRAF inhibitors. The new strategy is to combine BRAF and MEK inhibitors in first line therapy for BRAF-mutated patients. At 2011 ASCO Meeting, a trial combining a BRAF inhibitor (GSK2118436) and a MEK inhibitor (GSK1120212) was presented; it showed high response rates with a very good toxicity profile [37]. A similar ongoing trial is the BRIM-7, based on the combination of Vemurafenib and a MEK inhibitor (GDC-0973).

New possible combinations of multi-target drugs include MEKi, ERKi, PI3Ki, and AKTi. Ongoing trials are represented by: Phase Ib Study of PI3 (Phosphoinositol3)-Kinase Inhibitor BAY80-6946 with MEK (Mitogen-activated Protein Kinase) Inhibitor BAY86-9766 in Patients With Advanced Cancer (NCT01392521) and “A Study to Investigate Safety, Pharmacokinetics (PK) and Pharmacodynamics (PD) of BKM120 Plus GSK1120212 in Selected Advanced Solid Tumor Patients [NCT01155453]”. In the subset of ^V600E^BRAF-mutated population, the strategy of combining chemotherapic agents and small molecules, such as Levatinib or PARP Inhibitors, was adopted in order to overcome the hurdle of the less effective results of the chemotherapy.

In the BRAF-wild-type population, the principal strategy proposed for treating such patients in the future is the combination of chemotherapic agents and immunomodulating monocolonal antibodies. The comparison between the best overall response rate, disease control rate, and duration of response of the three randomized phase II-III studies with ipilimumab [38] showed how the combination of Chemotherapy and Ipilimumab is superior to Ipilimumab and Dacarbazine alone. The Phase II Study Combining Ipilimumab and Fotemustine in Patients with Metastatic Melanoma [NIBIT-M1 Trial] indeed demonstrated the advantage of this combination [39].

In both previously treated and non treated metastatic melanoma patients, albumin-bound paclitaxel (nab-paclitaxel) was well tolerated and showed a good activity in association with Carboplatin (4 months progression free survival, 74%; median progression free survival, 5.8 months).

Immunomodulating mAbs + Anti-angiogenetic compounds is another combination actually evalutated; as presented by Hodi at 2011 ASCO Meeting, the association of Ipilimumab with Bevacizumab gave interesting results in a small cohort of melanoma patients [40]. Furthermore, different immunomodulating antibodies may be combined in clinical trials. Associating two Immuno checkpoint blocking antibodies such as Ipilimumab and sub-efficacious doses of anti-PD1 was demonstrated to achieve a median reduction of the tumor volume much higher than that obtained using higher doses of the single antibodies in mouse models.

Finally, anti-CTLA-4 can be combined with either electrochemotherapy, through association of suboptimal doses of a chemotherapeutic agent - bleomicin or cisplatin - and an electroporation performed by an electrical impulse driven by a needle (interesting results were obtained at the National Cancer Institute of Naples as well as in other Institutions from different countries), or vaccination or T-reg depletors (as in the experience based on the use of Denileukin Diftitox).

Overall, several innovative weapons are available to fight melanoma; our efforts will be aimed at assessing the best strategy for the patients’ treatment. Surely, the motto in melanoma therapy for next years will be: Combine, Combine, Combine!

In patients with metastatic melanoma harboring V600 mutations GSK2118436 & GSK1120212 are both investigational agents, and the present standard of care is vemurafenib. In vemurafenib-refractory patients, or BRAF V600Wild-Type patients, the standard-of-care is either ipilimumab or high-dose IL-2 (in the U.S.) for those who did not receive these agents first-line, or chemotherapy for those who have received ipilimumab, IL-2 and vemurafenib. Considering the future development of investigational agents, possible phase III trial designs must consider the acheiveably endpoints (overall vs. progression-free survival) and the safety of the treatment in relation to the magnitude of benefit being sought. Using the example of GSK2118436 (BRAFi) and GSK1120212 (MEKi) the most scientifically rigorous control arm would be GSK2118436, whereas the conventional regulatory comparator would be vemurafenib. If the contribution of both agents to overall efficacy must be determined, then an additional control arm with GSK1120212 would be needed. Based on preliminary data with this two drug combination, the safety of the combination appears to be superior to either drug alone. If so, one might consider a lower threshold of increased efficacy to establish this combination as a new treatment standard then would be the case if the combination were more toxic than single agent therapy.

Unlike the example of GSK2118436 and GSK1120212, not all targeted or immunologic agents nominated as potential melanoma therapeutics are going to have single-agent activity; if synergistic, two agents should be active together even when neither is alone. Given that very few of the potential two drug combinations of investigational agents will arise from within a single pharmaceutical company, combining investigational agents early in clinical development involves significant risk-taking for the companies involved. Presuming that neither agent has significant single-agent activity, and independent approval may not be possible; having the success of one companies agent depend on the solvency of another company and willingness to invest in continued development of an agent lacking single agent activity calls for a greater degree of collaboration than has previously been manifested in the pharmaceutical industry. There is a need for increased infrastructure and a regulatory framework to facilitate investigational agents being combined early in development (such as NCI Cancer Therapeutics Evaluation Program). Moreover, companies are currently disincentivized to allow investigational agents to be combined with other investigational agents has unique toxicities observed with such a combination may hinder the development of each individual drug. Incentives must be created for the pharmaceutical companies to “contribute” agents into a pool of investigational agents.

Even among proven drugs, one can find examples where conflicting agendas might limit scientifically supported combination regimens. Treatment with a selective inhibitor of BRAF^V600E^ increases CD8+ T Cell infiltrate in tumors of patients with metastatic melanoma. This is likely a consequence of increased MDA expression with selective BRAF inhibitors when MITF expression is derepressed. These observations support the investigation of BRAF inhibitor/immunotherapy combinations and ipilimumab is a plausible agent for this purpose. Given that vemurafenib and ipilimumab are currently approved a single agents in metastatic melanoma and the pharmaceutical companies that produce them are vying for maximum market share, will the most scientifically rigorous clinical investigations be undertaken to evaluate this combination or inhibited out of concerns of new risks that could be uncovered which could taint the perceived safety profile of either agent?

Regulatory authorities must adapt to scientific underpinnings that drive the pursuit of combination therapies and maintain an awareness of the unmet need for the patient population and the line of therapy being investigated. Mechanism-of-action and clinical measures of benefit dictate optimal endpoints for definitive trials. Future advances will likely be limited by availability of investigational drugs for novel/novel combinations.

Heritable changes in the expression of single genes or patterns of genes not based on modifications of the DNA sequence are methylation in C5 of cytosine within CpG dinucleotides, hystone modifications and changes in chromatin structure. Hypomethylation generally result in gene expression while hypermethylation results in gene silencing. Epigenetic modifications are generally reversible pharmacologically as with Inhibitors of DNMT (e.g., 5-azacytidine, 5-aza-2′-deoxycytidine, Zebularine) or Inhibitors of HDAC (e.g., TSA, depsipeptide, SAHA,….). Epigenetically-regulated TAA in human cancer are MAGE-A1, -A2, -A3, -A4, -A6, -A10, MAGE-A12, BAGE, GAGE1-6, SSX1-5, NY-ESO-1, HAGE, PRAME, RAGE-1, etc. CTA expression is regulated by promoter methylation. CTA expression in melanoma cells can be regulated by DHA with a dose-dependent induction. Methylation statuses of melanoma cells may influence prognosis and (immune-)response to therapy. LINE-1 is a surrogate marker for global genomic methylation status, and, as shown by an analysis of 42 stage IIIC melanoma patients about survival according to LINE-1 methylation, hypermethylation is related with a poorer prognosis and specific methylation profiles associate with survival of stage IIIC melanoma patients. Instead LINE-1 methylation correlates with the number and level of expressed CTA.

The combination of IL-2 and standard doses of radiation has been tested in metastatic melanoma, with the conclusion that there is “. . . no apparent synergy in antitumor effect”. Stereotactic body radiation therapy (SBRT) is totally different from “conventional” radiation, because it uses multiple beams from multiple directions, achieving a higher dose to the tumor, lower dose to surrounding normal tissue and tumor motion (due to respiration) is taken into account using “4D planning” (a movie loop of CT images to determine treatment volume).

The rationale for testing SBRT + IL-2 is that high dose per fraction radiation, in contrast to standard dose fractions, can augment immune responses in murine tumor models by lowering intratumoral T_reg,_ increasing CD8 T-cell infiltration into the tumor, inducing antigen release, releasing Damage-Associated Molecular Patterns (DAMPs), HMGB1 and up-regulating MHC class 1, B7.1 and Fas/CD95. IL-2 can induce clinically meaningful immune responses in (a minority of (ORR ~15–16%)) patients with metastatic melanoma and renal cancer.

A phase I dose-escalation study of SBRT was performed in patients with widely metastatic melanoma to determine the maximum tolerated dose of SBRT when used in conjunction with high-dose IL-2. The study measured the local control of SBRT-treated lesions, estimated the overall tumor response, and to monitored toxicities. Exploratory studies of immune responses on peripheral blood mononuclear cells were also performed using polychromatic flow cytometry.

5 out of 7 patients with melanoma had objective regression. All SBRT-treated lesions regressed and there were some responds in lesions not treated with SBRT. There no dose-limiting toxicities from SBRT and the IL-2 toxicities were those anticipated. All 5 patients had a complete regression of melanoma by PET imaging, although minor residual imaging abnormalities persisted on CT in 4 of these patients.

Responding patients showed increased proliferation at baseline and after IL-2 of CD4+ T cells with activated T_EM_ phenotype (CD25^+^FoxP3^-^Ki67^+^CCR7^-^CD45RA^-^CD27^+^CD28^+/−^) and CD8+ T cells with early T_EM_ phenotype (Ki67^+^CD25^-^CCR7^-^CD45RA^+^CD27^+^CD28^+^). There were no change in proliferation of T_reg_ comparing responders and non-responders.

## Competing interests

PAA participated to Advisory Board from Bristol Myers Squibb, MSD, Roche-Genentech, GSK, Celgene, Amgen, Medimmune, and Novartis and received honoraria from Brystol Myers Squibb, MSD and Roche-Genentech.

AMG has no competing interest. BC Prometheus Pharmaceuticals: grant support and speakers bureau. MBF has no competing interests. SF has no competing interests. KF Consultant: Roche/Genentech, GlaxoSmithKline. BAF has no competing interests. TG consultant for GSK-Bio, Incyte, BMS, Roche-Genentech, and Eisai. JEG has no competing interest. HG has an Advisor role in MSD compensated. KG has participated on Advisory Board for BMS, and has received honoraria from BMS. AH consultancies, paid presentations or financial trial support from: BMS, Boehringer Ingelheim, Celgene, Eisai, GSK, IGEA, MSD, Novartis, Roche-Genentech. SH has served as a nonpaid consultant to BMS and Genentech, and received clinical trial support from BMS and Genentech. RK Institutional reimbursement for Advisory Boards and conference travel from GSK and Roche. JMK is a consultant to GSKbio, and has participated in advisory boards for Novartis, Merck, and GSK. SL serves on an Advisory Board for Myriad Genetics Laboratories. MM Advisory Boards from BMS, Roche and GSK. RM receives research support from Novartis, and has consulted for Roche. He is eligible to income from drugs that are commercialized by the Institute of Cancer Research through the “Rewards to Inventors Scheme”. DLM has no competing interest. GP has no competing interest. AR participated to Advisory Board from Amgen, Bristol Myers Squibb, Celgene, Roche-Genentech, GSK, Merck, Millennium, Novartis and Prometheus and received honoraria from these companies. DFS is a co-inventor on a patent related to the growth of TIL in gas permeable flasks. RS has no competing interest. EW has no competing interest. NM has no competing interest. FMM has no competing interest.

## Authors’ contributions

PA, AMG, and DFS prepared the manuscript collaboratively with input and review by all co-authors. All authors read and approved the final manuscript.
